# Supplementary material for: Prevalence and risk factors associated with intestinal parasitosis in children with diarrhoea in the Huambo municipality
Source: PLoS Negl Trop Dis. 2025 Jul 21;19(7):e0013314. doi: 10.1371/journal.pntd.0013314 (PMC12393785; doi:10.1371/journal.pntd.0013314)
Supplement: Attachment — Submitted filename: Comments on Manuscript PNTD-D-24-01894.pdf [file pntd.0013314.s002.pdf]

1. Recommendation term: **Minor Revision.** The study is well-structured and addresses an important public health issue. However, minor concerns related to methodology clarity, statistical interpretation, and English language improvements should be addressed before acceptance.

2. Methods:

- Are the objectives of the study clearly articulated with a clear testable hypothesis stated?

Yes, the objectives are clear, but a specific hypothesis is not explicitly stated. It would be beneficial to include a testable hypothesis in the introduction.

- Is the study design appropriate to address the stated objectives?

Yes, the study design is appropriate for determining prevalence. However, additional justifications regarding sample selection and potential biases would improve the robustness.

- Is the population clearly described and appropriate for the hypothesis being tested?

Yes, the population (children aged 9 months to 14 years hospitalized with diarrhea) is clearly described and appropriate for the study objectives.

- Is the sample size sufficient to ensure adequate power to address the hypothesis being tested?

The sample size (173 children) seems reasonable for a prevalence study, but the authors do not provide justification for the sample size.

- Were correct statistical analyses used to support conclusions?

Yes, the statistical analyses (descriptive statistics, Chi-square test, Odds Ratio) are appropriate for analyzing risk factors. However, some p-values are close to 1, raising concerns about statistical power.

- Are there concerns about ethical or regulatory requirements being met?

Ethical approval is mentioned, and informed consent was obtained. No major concerns in this area.

3. Results:

- Does the analysis presented match the analysis plan?

The results align with the methodology, but some statistical findings need clearer interpretation (e.g., why none of the risk factors were statistically significant despite apparent trends).

- Are the results clearly and completely presented?

The results are generally well-presented, though some tables and figures (e.g., Figure 1) would benefit from clearer labeling and descriptions.

- Are the figures (Tables, Images) of sufficient quality for clarity?

Tables and figures are informative, but figure quality should be improved for readability.

4. Conclusions:

- Are the conclusions supported by the data presented?

The conclusions are mostly supported, but the claim that risk factors were not statistically significant could be better contextualized.

- Are the limitations of analysis clearly described?

The limitations (e.g., diagnostic method sensitivity, sample size, geographic restriction) are clearly described, but more discussion on potential confounders (e.g., socioeconomic status) would be beneficial.

- Do the authors discuss how these data can be helpful to advance our understanding of the topic under study?

Yes, the authors discuss the public health implications and the need for further research and interventions.

- Is public health relevance addressed?

Yes, the public health relevance is addressed, particularly in terms of the need for deworming programs and hygiene education.

5. Editorial suggestions or minor modifications:

- The authors should consider adding a hypothesis statement in the introduction to clarify the study’s aims.

- The authors should use italics for scientific names
- Provide a stronger justification for the sample size.
- The study did not report any cases of polyparasitism (co-infections with multiple parasites), which is somewhat surprising given the context. This could be due to the limitations of the diagnostic method used.
- Enhance figure clarity with better labels and descriptions.
- While the authors analyzed various risk factors (e.g., age, sex, water source, hygiene practices), none were found to be statistically significant. Clarify why statistical significance was not observed despite apparent trends in the data.
-

6. Confirmation of comments for authors:

All comments are suitable for the authors to view.

7. Competing interests:

No competing interests.

8. Ethical standards:

Yes, the research appears to follow high ethical standards.

9. Concerns if "No" to ethical standards:

N/A

10. Data availability:

The authors state that the data are available in the hospital database in Angola, but they do not provide a specific link or repository. It would be helpful if they could make the data more accessible, possibly through a public repository.

11. Intelligible and standard English:

The manuscript is understandable but needs English language refinement.

12. Additional copyediting:

Professional copyediting would improve readability.

13. Identify yourself to the authors and other reviewers:

No

14. Recognition for this review on Publons:

Yes
